# Bone Regeneration, Reconstruction and Use of Osteogenic Cells; from Basic Knowledge, Animal Models to Clinical Trials

**DOI:** 10.3390/jcm9010139

**Published:** 2020-01-04

**Authors:** Greg Hutchings, Lisa Moncrieff, Claudia Dompe, Krzysztof Janowicz, Rafał Sibiak, Artur Bryja, Maurycy Jankowski, Paul Mozdziak, Dorota Bukowska, Paweł Antosik, Jamil A. Shibli, Marta Dyszkiewicz-Konwińska, Małgorzata Bruska, Bartosz Kempisty, Hanna Piotrowska-Kempisty

**Affiliations:** 1 The School of Medicine, Medical Sciences and Nutrition, Aberdeen University, Aberdeen AB25 2ZD, UK; g.hutchings.16@abdn.ac.uk (G.H.); l.moncrieff.16@abdn.ac.uk (L.M.); claudia.dompe.16@abdn.ac.uk (C.D.); krzysztof.janowicz.16@abdn.ac.uk (K.J.); 2 Department of Anatomy, Poznan University of Medical Sciences, 60-781 Poznan, Poland; abryja@ump.edu.pl (A.B.); mjankowski@ump.edu.pl (M.J.); m.dyszkiewicz@ump.edu.pl (M.D.-K.); mbruska@ump.edu.pl (M.B.); 3 Department of Histology and Embryology, Poznan University of Medical Sciences, 60-781 Poznan, Poland; 4 Division of Reproduction, Department of Obstetrics, Gynecology, and Gynecologic Oncology, Poznan University of Medical Sciences, 60-535 Poznan, Poland; 75094@student.ump.edu.pl; 5 Physiology Graduate Program, North Carolina State University, Raleigh, NC 27695, USA; pemozdzi@ncsu.edu; 6 Department of Diagnostics and Clinical Sciences, Nicolaus Copernicus University, 87-100 Torun, Poland; dbukowska@umk.pl; 7 Department of Veterinary Surgery, Nicolaus Copernicus University, 87-100 Torun, Poland; pantosik@umk.pl; 8 Department of Periodontology and Oral Implantology, Dental Research Division, University of Guarulhos, 07030-010 Guarulhos, SP, Brazil; jashibli@yahoo.co; 9 Department of Biomaterials and Experimental Dentistry, Poznan University of Medical Sciences, 60-812 Poznan, Poland; 10 Department of Obstetrics and Gynecology, University Hospital and Masaryk University, 601 77 Brno, Czech Republic; 11 Department of Toxicology, Poznan University of Medical Sciences, 61-131 Poznan, Poland; hpiotrow@ump.edu.pl

**Keywords:** bone, regeneration, reconstruction, osteogenesis, stem, cells

## Abstract

The deterioration of the human skeleton’s capacity for self-renewal occurs naturally with age. Osteoporosis affects millions worldwide, with current treatments including pharmaceutical agents that target bone formation and/or resorption. Nevertheless, these clinical approaches often result in long-term side effects, with better alternatives being constantly researched. Mesenchymal stem cells (MSCs) derived from bone marrow and adipose tissue are known to hold therapeutic value for the treatment of a variety of bone diseases. The following review summarizes the latest studies and clinical trials related to the use of MSCs, both individually and combined with other methods, in the treatment of a variety of conditions related to skeletal health. For example, some of the most recent works noted the advantage of bone grafts based on biomimetic scaffolds combined with MSC and growth factor delivery, with a greatly increased regeneration rate and minimized side effects for patients. This review also highlights the continuing research into the mechanisms underlying bone homeostasis, including the key transcription factors and signalling pathways responsible for regulating the differentiation of osteoblast lineage. Paracrine factors and specific miRNAs are also believed to play a part in MSC differentiation. Furthering the understanding of the specific mechanisms of cellular signalling in skeletal remodelling is key to incorporating new and effective treatment methods for bone disease.

## 1. Introduction

Skeletal homeostasis, bone repair and regeneration in the adult skeleton are complex processes, with a significant amount of research currently focusing on these aspects. The three main cell types involved in this process—osteoblasts, osteoclasts and osteocytes—interact via signalling molecules to regulate the differentiation of progenitor cells to model and remodel the skeleton [1]. Mesenchymal stem cells (MSCs) found in bone marrow hold the potential to differentiate into osteoblasts, which then mature into osteocytes, a process initiated by the release of transforming growth factor-β1 (TGF-β1) from neighbouring osteoclasts [1]. The imbalance and dysfunction of the bone remodelling cycle is the basis for a variety of bone disorders, including osteoporosis and osteosclerosis [2].

The implantation of MSCs has already demonstrated therapeutic value, increasing osteointegration in conjunction with modern tissue engineering with synthetic scaffolds [2,3,4,5]. Furthermore, the addition of specific growth factors can improve the healing rate and effectiveness of treatment [6]. Technologies such as centrally vascularized tissue engineering bone grafts (CV-TEBG) use bone and blood vessel progenitor cells to promote osteogenesis and vascularization at the scaffold site [7].

However, before advanced stem cell treatments for a variety of conditions can be implemented, a thorough understanding of cell lineage and the regulation of differentiation by transcription factors and signalling pathways is required. Three key transcription factors–Runt-related transcription factor 2 (RUNX2), Osterix/Sp7 (Osx) and Dlx5—are known to play an important role in the osteogenic differentiation of MSCs. RUNX2 and Osx act as key regulators to both activate and inhibit differentiation at different cell maturation stages [8,9]. These factors regulate multiple signaling pathways involved in MSC differentiation, including Akt, BMP, IGF and Wnt [10,11,12]. Wnt signaling is known to play a central role in osteogenic differentiation, with its natural age-associated decrease leading to the dysfunction of bone regeneration [12]. Notably, the delivery of nanoparticles could activate Wnt signalling in mesenchymal stem cells [13].

MSCs have already demonstrated a significant therapeutic potential in a variety of clinical trials, e.g., in the healing of non-union fractures in mice [14]. Furthermore, adipose tissue-derived mesenchymal stem cells (ASCs) show great promise as an alternative source of osteogenic cells for treatment. Similarly, MSCs delivered through the injection of micro-fragmented adipose tissue may restore cartilage function, inducing the interconversion of adipocytes by an adipose tissue carrier [15,16]. Such modifications to osteocyte–adipocyte balance could serve to supplement future treatments of osteoporosis, as well as other related diseases [2]. Moreover, a 2019 study suggested that bone marrow concentrate may hold potential for more effective treatment than MSCs alone, due to crosstalk between MSCs and other progenitor cells [17].

An improvement in the understanding of osteogenic stem cell differentiation, including the factors, signalling pathways and small molecules involved in this process, could result in novel therapeutic opportunities of bone disease treatment. Moreover, current techniques could be enhanced through the analysis of cellular communication in skeletal tissue during repair and remodelling, in both normal and pathological conditions [17,18]. Hence, this review summarizes the newest findings on the potential and current research associated with the clinical applications of cells with osteogenic differentiation potential.

## 2. Section I: Current State of Knowledge on Bone Regeneration—Tissue and Cell Specific Mechanisms

The homeostasis of the human skeleton is maintained and regulated by complex interactions between osteoblasts, osteocytes and osteoclasts. Osteoblasts are active in protein synthesis and matrix secretion to maintain and form new healthy bones, while osteoclasts are responsible for bone resorption in repair and development. Following matrix mineralization, fully matured osteoblast cells become osteocytes, and are imbedded in the matrix surrounding the cell. In bone modelling, i.e., during development, bone formation and resorption, osteocytes act independently to alter bone structure. Conversely, in bone remodelling, formation and resorption, they act in tandem to repair and maintain skeletal health, without affecting bone shape [19]. Although detailed molecular mechanisms of these processes remain to be uncovered, osteocytes are known to directly and indirectly (e.g., through the regulation of Wnt signalling pathways) regulate the activity of osteoblasts and osteoclasts [20,21].

### 2.1. The Sources, Differentiation Pathways and Stemness Potency of Tissue-Derived Osteogenic Cells

Adult bone marrow is known to contain mesenchymal stem cells, which are able to differentiate into a range of cell lineages, forming mesenchymal tissues such as bone, cartilage and muscle. The bone marrow stem cell population can be divided into hematopoietic and stromal cells, both of which show multipotency and high proliferative potential.

Although the characterization of bone marrow stem cells (BMSCs) is quite controversial in the current literature [22], populations of cells exhibiting osteogenic potential were obtained through the isolation of non-plastic adherent bone marrow cells and administered to the human peripheral circulation to improve bone fracture repair [23,24]. Traditionally, the identification of BMSCs, which were also observed in small numbers in the circulation, is determined by their adherence to plastic. However, it is also possible using CD271 as a specific marker [25]. MSCA-1 and SUSD can also be used as BMSC markers, but are not effective in MSCs obtained from the adipose tissue, which can be isolated through the positive expression of CD34^+^ [26]. Moreover, bone marrow osteogenic cells without plastic adherence can be identified through the positive expression of bone-specific alkaline phosphatase and/or osteocalcin [24].

Three transcription factors have been found to play important roles in MSC osteogenic differentiation potential. These are Runt-related transcription factor 2 (Runx2), Osterix (Osx) and Dlx5. Runx2/Cbfα1 is observed to have two main roles—the promotion of osteogenesis and the inhibition of adipocyte differentiation [27]. Chava et al. discovered that Runx2 is a substrate of AMP-activated kinase, with a lack of phosphorylation at serine 118 residue in the DNA-binding domain of Runx2 leading to adipogenesis, negating the osteogenic potential of MSCs [27]. Furthermore, Runx2 is essential for osteoblast differentiation, with its activation influencing several steps of this process. Runx2 activation in stem cells results in their definition as pre-osteoblasts, initiating the differentiation process [18]. Each of the three steps of differentiation are defined by the expression of different molecular markers, often regulated and induced by Runx2, including [28]. Upon commitment to the osteogenic lineage, the expression of genes supporting proliferation, cell cycle progression and extracellular matrix biosynthesis significantly increases. Later, the genes responsible for proliferation are subject to repression, coupled with the further upregulation of those involved in the maturation and organisation of the extracellular matrix [18]. The involvement of Runx2 in the differentiation of MSCs into osteoblasts was firstly demonstrated by Komori et al. in 1997. In this mouse model study, low concentration Runx2 led to the impairment of bone formation. The activity of Runx2 is time- and concentration-dependent. Its levels are elevated at the point of stem cell commitment to the osteogenic lineage, during which it promotes osteogenesis. In the later stages of osteoblast maturation, its levels are downregulated, until the point of total absence during final osteocyte differentiation [8]. Moreover, in these later stages of differentiation, higher Runx2 levels have an inhibitory effect, resulting in reduced bone mass [29]. Furthermore, Runx2 plays a big role in the activation and enhancement of other signalling pathways, being involved in various physiological processes. As an example, Runx2 was observed to interact with the PI3K-Akt signalling pathway in a mutually dependent manner. Runx2 upregulates PI3K-Akt, which in turn promotes DNA binding to Runx2, as well as Runx2-dependent transcription [30].

Over 10 years ago, Osx, a zinc finger-containing transcription factor, was first identified. Studies of its functions led to the conclusion that it is involved in osteogenesis, not only promoting primary crystal formation and the commitment of stem cells to the osteogenic lineage, but also playing an important role in bone homeostasis and inhibiting later stages of osteogenic differentiation. In fact, Osx exhibits a similar mode of inhibition in the later osteogenic differentiation stages as Runx2, with its upregulation maintaining osteoblasts at an immature state [9]. A 2002 study by Nakashima et al. showed no mutual dependence between these two proteins at this stage, as opposed to other studies suggesting that Osx activation is dependent on Runx2 [9,31].

Moreover, in Osx-null embryos with a regular expression of Runx2, low to no expression of osteoblast markers was observed [31]. Finally, a study by Nishio et al. proved that Osx lies downstream of Runx2, showing that no Osx expression was observed in Runx2-null embryos, but the lack of Osx does not impact Runx2 expression [32]. 

Regarding the expression of Osx and its osteogenesis upregulating activity, multiple signalling pathways were found to be involved in this process. BMP2 and IGF-1 were shown to work synergistically in the Osx upregulation. IGF-1, in a sheep model study, showed the ability to promote osteogenesis during bone regeneration, with its expression inducing osteoblast differentiation [33]. IGF-1 was shown to promote early stages of differentiation, acting through the Akt/MAP kinase pathway, with its concentration observed to be very low in later osteogenic phenotypes [10]. In contrast, BMP2 was observed to promote osteogenesis in a Runx2-dependent manner, with its expression not requiring all three MAPK components (Erk, p38, and JNK), relying only on p38 and JNK. Moreover, the combined expression of BMP2 and IGF1 further promotes osteoblast differentiation, working through the MAPK and PKD pathways [11]. On the other hand, osteoblast gene expression is negatively regulated by EGFR and TNF. Through the inhibition of Runx 2 through HDAC stimulation, EGFR has a downregulating effect on Osx [34]. Furthermore, different histone deacetylases were also found to have a role in this process [35,36,37]. TNF alpha upregulates Prx1, which represses the expression of Osx [38]. 

Moreover, Osx plays an important role in bone homeostasis, influencing the formation of bones and the expression of osteoblast genes in adults. A lack of Osx expression led to the absence of endochondral ossification and errors in cartilage matrix ossification and matrix vesicle formation [39]. In fact, synergistically with Runx2, Osx was observed to have a few binding sites on ectonucleotide pyrophosphatase/phosphodiesterase 1 (ENPP1). ENPP1 is responsible for bone mineralization and was described to be upregulated by Osx through p38-mitogen-activated protein kinase [40]. Another activity of Osx that restrains bone mineralization is the activation of sclerostin, which is a Wnt pathway antagonist, inhibiting bone formation [41].

**Figure 1:**
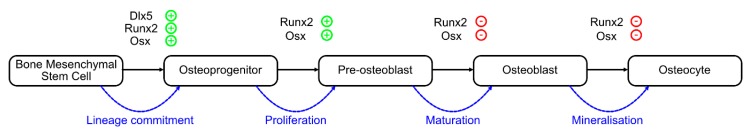
Differentiation pathway of the osteoblast lineage. Lineage commitment occurs through mesenchymal stem cell (MSC) to osteoprogenitor transition. Proliferation is the next stage, during which the pre-osteoblast is formed. The maturation of the pre-osteoblast is followed by the mineralization of the matrix, in which the fully matured osteocyte embeds. Dlx5, Runx2 and Osterix (Osx) are key transcription factors which play the roles of both activators and inhibitors (+ or −) during different stages of differentiation and maturation.

Dlx5 is yet another transcription factor involved in osteogenesis, with studies of Dlx5-null mice showing a range of defects [42,43]. Dlx5-null mouse embryos showed lowered bone volume, as well as lowered osteoblast proliferation and differentiation, due to a lower expression of Runx2, Osx, Osteocalcin and bone sialoprotein. Moreover, the absence of Dlx5 leads to an increase in osteoclast number, suggesting that Dlx5 may regulate osteoblast differentiation to osteoclast, as osteoclasts do not express this transcription factor. Dlx5 was also shown to be an activator of Runx2, with Dlx5 responsive elements identified in the bone-specific P1 promotor of Runx2 [44,45]. However, Dlx5 promotes the expression of ALP and osteocalcin in Runx2-null mice [44], meaning that it can also work in a Runx2-independent manner [46], which can similarly lead to the activation of Osx [47]. In particular, Dlx5 is already expressed at very early stages of bone development and has been proposed to play a central role in the control of osteogenesis. [48] It was proven that DlX5 acts at other stages of osteogenesis, controlling the expression of Osteocalcin and BSP, which are expressed in the differentiated osteoblast, as they are both downregulated in Dlx5-null mice. The absence of Dlx5 in mice caused the impairment of bone homeostasis and osteoblast/osteoclast coupling. Moreover, Dlx5 is upregulated by BMP2 [49], mediating BMP2-induced Runx2 expression [50]. A graphical representation of the osteoblast differentiation pathway is presented in Figure 1.

Transcription factors from the AP1 family were described to promote osteogenesis, being observed during proliferation and absent during differentiation [51]. C-Fos, for example, was observed to have no effect on bone formation but its overexpression resulted in the osteosarcoma phenotype [52]. Moreover, gain-of-function studies of Fra and ΔFosB, the overexpression of which led to osteosclerosis, showed enhanced osteoblast formation [53,54]

Osteoclast formation not only depends on the interaction of many transcription factors and pathways, but also, as proved by in vitro studies, on the communication between precursor cells and already differentiated osteoblasts [55]. M-CSF is an essential osteoclast survival and proliferation factor [56]. Nevertheless, mice studies showing that the deficiency of other factors also caused osteoporosis led to the discovery of other molecular pathways influencing osteoclastogenesis, with Nuclear factor kappa-β ligand (RANKL) recognized as the most important differentiation factor [57]. Many more genes and transcription factors that influence differentiation of osteoclasts were discovered afterwards, such as TRAF6 [58], c-Fos [59,60], Nf-kB [61], NFATc1 [62] and FcRy/DAP12 [63]. 

As previously mentioned, while RANKL–RANK signalling is responsible for differentiation, M-CSF is essential for the proliferation and the survival of the osteoclast precursors [64]. Binding to its membrane receptor, it recruits Grb2 and src, which induce the ERK and PI3K pathways. When RANKL binds to its membrane receptors, it leads to the activation of numerous pathways involved in osteoclast differentiation, including NFATc1. Moreover, osteoclast precursors need costimulatory signals for the activation of differentiation processes. Ig-like ligands are expressed on osteoclast precursors and as costimulatory molecules to the RANK signalling [65].

M-CSF is in charge of the survival of the osteoclast precursor, also influencing the late stages of differentiation through the akt, c-Fos and ERK pathways [56]. Moreover, RANK recruitment of TRAF6 was proven to be very important for osteoclast differentiation, leading to the activation of NK-KB and MAPK pathways [66,67]. There is a range of proteins in the NF-KB family of transcription factors but only two were observed to impact bone formation, as mice lacking p50 and p52 showed defects due to impairments in DNA binding [61]. Further studies led to the conclusion that only the classical NF-Kb pathway activation, involving the activation of the kB kinase, has a negative impact on osteoclastogenesis [68,69]. NFATc1 is a target gene of NF-Kb, which is recruited to NFATc1 promoter when RANKL is stimulated [70]. 

Another important osteoclast differentiation factor belonging to the AP-1 family is c-Fos, with studies showing that mice lacking this protein developed osteoporosis [60,71]. In vitro studies of MAPK showed the importance of this pathway in osteoclastogenesis, as it leads to the activation of AP-1 components [72]. NFATc1 has an essential role in auto-amplification, not only due to its function, but also because of its gene regulatory mechanism. In fact, NFATc1 regulates TRAP, calcitonin receptor, cathepsin K and OSCAR, osteoclast-specific immunoreceptor osteoclast-associated receptor [73], with these genes activated by the transcriptional complex formed by NFATc1 with PU.1, AP-1 and MITF [62]. 

**Figure 2:**
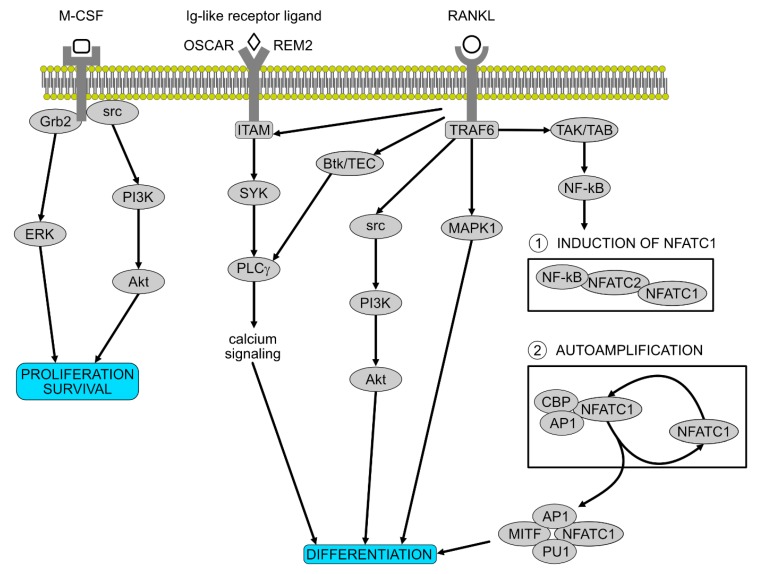
Molecular pathways involved in osteoclast proliferation and differentiation. M-CSF is essential for the proliferation and the survival of the osteoclast precursors. After activation, it recruits Grb2 and src which induce ERK and PI3K pathways. RANKL is the most important differentiation factor, activating numerous pathways involved in osteoclasts differentiation, including the two stages governed by NFATC1. Ig-like receptor ligands on osteoclast precursors synergize with the RANK signalling.

The selection of an appropriate source of skeletal progenitors is important for skeleton regeneration therapies. Mesenchymal progenitors isolated from long bone regions showed a similar cellular morphology and marker expression but different proliferation and differentiation potential [74]. For example, cells isolated from the endosteum showed a higher capacity for proliferation, differentiation and metabolic activity compared to bone marrow mesenchymal progenitors. In fact, they showed an increased osteoclast formation ability [74]. Periosteum is also a potential source of cells of osteoblastic lineage. It has been used in a wide variety of orthopedic surgeries and has proven promising, promoting bone growth and repair in vivo [75]. Periosteum-derived progenitor cells (PDPCs) exhibit multipotency at the single cell level, high proliferation and differentiation rate in vitro, as well as similar potency when isolated from young and old patients [76,77]. However, although metabolic activity is maintained, PDPC proliferation declines with senescence [78]. The dentification of periosteum MSCs is still questionable, as a specific marker has not currently been found. Periosteum is responsible for contributing to the callus formation, participating in intramembranous and endochondral ossification upon fracture [79]. PDPCs express chemokine receptors which respond to the stromal cell-derived factor 1 (SDF-1) and B cell-attractive chemokine (BCA1), which are both expressed in osteoblasts obtained from post-traumatic or osteoarthritis patients [80]. This shows the potential of PDPCs both as a chemoattractant and as a signalling molecule for in situ bone regeneration. Although in periosteal cells near the site of injury, SDF-1 is observed to be upregulated and attracts MSCs expressing CXCR4, the influences of SDF-1 and CXCR4 in the differentiation potential need to be further investigated [80]. BMP2 is also involved in the proliferation and differentiation of periosteal progenitors, as in vivo studies show a lack in healing in the absence of this protein [81,82]. Studies have shown how applying tension in periosteum, which due to its localization is sensitive to mechanical stimuli in its native state, promotes bone healing after surgery [83]. Moreover, periosteum homeostasis and regeneration are regulated by macrophage-lineage cells, which recruits periosteum-derived cells for the formation of cortical bone [84]. The overview of molecular pathways involved in osteoclast proliferation and differentiation has been presented in Figure 2**.**

In summary, further research is needed to clarify the signalling pathways related to BMP2, to allow the development of new targets to promote osteochondral repair. Furthermore, to characterize the mechanisms involved in the differentiation of pre-osteoblasts, it would be fundamental to collect more data about miRNA expression in the periosteum. 

### 2.2. The Aging of the Skeleton

The capacity of the human skeleton for self-renewal diminishes severely with age. Osteoporosis, characterized by the loss of bone mineral density and bone strength, primarily affects older people and is more likely to affect women than men. It is estimated that over 200 million people worldwide are affected by osteoporosis, with 1 in 3 women over the age of 50 likely to be suffer fractures [85]. An early-onset version of osteoporosis is known to be caused by an autosomal dominant mutation in the *Wnt1* gene [86].

Age-related reduction in bone healing ability is caused by an increasing imbalance of bone formation and resorption. Current osteoporosis treatments include pharmacological agents which attempt to slow down bone resorption, including bisphosphonates and/or estrogenics, or increase bone formation via parathyroid hormone peptides [85,87]. Combined with these treatments, early screenings for those most at risk combined with the lowering of risk factors due to lifestyle, such as tobacco smoking, alcohol intake and poor diet, is recommended. Furthermore, novel treatments based on inhibitors of bone resorption or stimulators of bone formation can target bone resorption/formation independently, effectively uncoupling these processes and leading to more efficient and effective treatment [87]. The long term use of these medications has been linked to impairments in bone microarchitecture, as well as a rebound effect following the discontinuation of drug use, increasing the risk of multiple fractures [88,89]. Therefore, the challenge remains to find safe, effective treatments which minimize secondary risks.

Cathepsin-K (CatK), expressed in osteoclasts, is a cysteine protease important to bone resorption, particularly the breakdown of bone collagen. Notably, a rare hereditary disorder known as pycnodysostosis can occur when the *CatK* gene is mutated, causing cathepsin-K deficiency and manifesting as the high bone density phenotype [90]. The CatK inhibitor odanacatib showed potential as a therapeutic agent to reduce bone resorption; however, following a link to increased risk of stroke in clinical trials, production of the drug was discontinued as of 2016 [88,90]. 

A tendency of MSCs in the body to favour an adipocyte lineage over osteoblast increases with age [2,91]. This could be caused by the downregulation of osteogenic genes—*Runx2* and *BGLAP*—and an upregulation of adipogenic genes—*PPAR-γ* and *aP2* [92]. Also important to note is the age-related decrease in efficacy of Wnt signalling, leading to reduced repression of *Sox2*, and subsequent differentiation of skeletal stem cells to chondrocyte over osteoblast lineage [12]. A detailed understanding of the mechanisms of MSC differentiation are necessary before implementation of new treatment methods is plausible.

## 3. Section II: Therapies

### 3.1. MSC- and ASC-Based Cell Therapies

**Figure 3:**
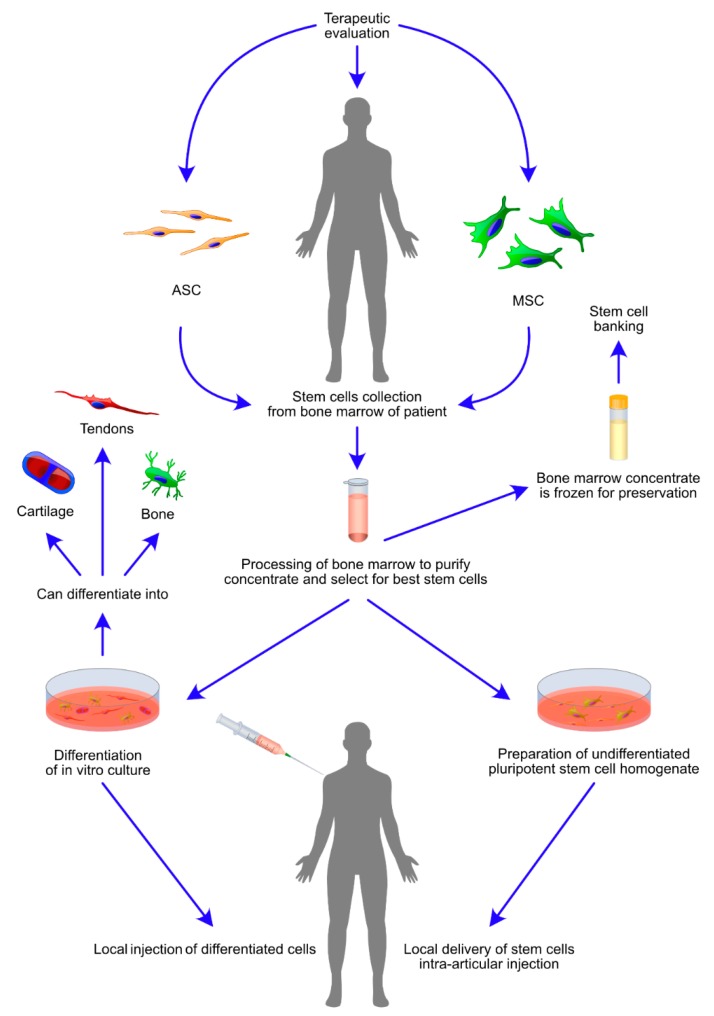
Therapeutically relevant events in administration of highly osteogenic mesenchymal stem cells (MSCs) and adipose stem cells (ASCs) for the treatment of osteogenic diseases. The schematic diagram presents steps crucial for intra-articular injection of bone marrow derived stem cells (BMSCs). The figure further defines two routes of therapy delivery, first: stem cells are in vitro cultured and differentiated using conditioned medium, followed by local delivery; second: stem cells are intra articularly administered in an uncultured and undifferentiated form. The local delivery in both cases is intra-articular or performed at the site of bone defect or fracture. Clinical routine favours administration of MSCs and ASCs as soon as the stem cell homogenate is processed. However, it is also possible to freeze the cells for a further administration. The diagram is supplemented by graphical representation of potentially existing differentiation outcomes of bone marrow stromal cells (BMSCs), including the formation of bone, cartilage and tendons.

MSC-based cell therapies focus on the improvement of osteoinduction through supplementation of tissue engineered construct (TEC) with isolated mesenchymal stem cells cultured in osteogenic medium [93]. The success of all clinical studies using MSCs and ASCs in bone regeneration depends on cell viability, cell homing capacity and engraftment into injured tissue [94]. Allogenic mesenchymal stem cells have been noted to achieve therapeutic effects in in vivo mouse studies investigating non-union bone [14]. The immunosuppression of effector functions, preventing the development of immunological diseases, is possible due to the communication of innate and adaptive immune cells with MSCs via inflammatory cytokines e.g., IL17, IL-6, IFN-γ and growth factors e.g., hepatocyte growth factor (HGF), prostaglandin E2 (PGE2), transforming growth factor-β (TGF-β) [95]. Furthermore, the inhibitory effects of MSCs on the proliferation of T cells are dependent on the expression of interleukin 17 (IL-17), such that in mice, IL-17-induced mesenchymal stem cells had their homing ability enhanced, resulting in the prolonged survival time of allogeneic skin grafts through suppressed immune recognition [96]. Interleukin IL-6 stimulates the proliferation and survival of T cells, in contrast to interferon-γ (IFN-γ), which suppress the proliferation of regulatory T cells and other immune cells [97]. MSC-secreted cytokines are required for MSC-mediated immunomodulatory effects, with better results observed in viable MSCs due to their intact secretome [98]. Moreover, proliferation and differentiation processes also affect the immunomodulatory effects of MSC-based cell therapies and influence the success of MSC transplantation, minimising the risk of graft rejection [99]. The results of knockdown studies reported that regulatory functions of either CD4+KO, CD25+KO or Foxp3+KO T regulatory cells were rescued by the presence of TGF-β1 secreting MSCs; a phenomenon present in myelodysplastic syndrome, where the elevation of TGF-β1, associated with cellular apoptosis, has similar effects [100]. A new approach to complex fractures and critical bone defect repair has recently been established, basing on the intra-articular injection of modular microtissues containing undifferentiated mesenchymal stromal cells associated with the chitosan–collagen matrix [101]. A few years ago, Tohma et al. validated the low osteogenic ability of allogenic bone grafts to be enhanced in cultures of Fischer 344 rat bone marrow autologous MSCs seeded on irradiated allogenic bone [102]. The influence of MSC and ASC administration on BMSCs was investigated by exposing allogenically activated lymphocytes to either MSCs or ASCs before and after osteogenic induction in vitro. This comparison demonstrated the immunosuppressive role of MSCs and ASCs on proliferative behaviour of allogenic peripheral blood mononuclear cells (PBMSCs) [103]. The usual steps associated with the clinical use of MSCs are graphically presented on Figure 3.

### 3.2. Advances in Bone Reconstruction Strategies

Current techniques of bone repair and regeneration in cases of non-union fracture or bone defects include autologous bone grafts, and require consideration of osteoinduction, osteoconduction, but also vascularization of the damaged area. Resident osteoprogenitor cells are always located close to small blood vessel networks, which play a key role in bone formation and remodelling. In endochondral bone ossification, hypertrophic chondrocytes release vascular endothelial growth factor (VEGF) and fibroblast growth factors (FGF) to stimulate angiogenesis [104]. Additionally, during bone remodelling, blood vessels are responsible for the transport of precursor cells to sites of bone resorption or formation. Autografts, usually taken from the iliac crest, must therefore allow the growth of new blood vessel networks while minimizing inflammation. With increasing age, the efficacy of autografts diminishes, along with the capacity of the skeleton for self-renewal. Recently, this has been shown to be linked to reduced endogenous Wnt signalling in aged animals. The promotion of Wnt signalling caused a significant increase in autograft healing potential in mice, highlighting a potential therapeutic target in bone health [105]. 

Alternative therapies, such as bioengineered scaffolds, have recently been developed, eliminating complications, such as donor site morbidity arising from autografts. Originally, these scaffolds were designed to match the physical properties of the tissue while remaining inert to the microenvironment. Following implantation, a non-specific immune response would eventually result in the scaffold being encapsulated by connective tissue [106]. More recently, biomimetic scaffolds have advanced to mimic autografts, and are supplemented with MSCs, endothelial cells and growth factors to increase the osteoconductive, osteoinductive and angiogenic properties of the repair site [5,106]. 

MSCs may regulate the proliferation of major immune cells and thereby play an important immunoregulatory role during implantation [107]. MSCs have also been shown to express low levels of MHC (major histocompatibility complex) type I molecules and no MHC type II molecules, allowing the avoidance of certain mechanisms of immune rejection by the host [108].

#### 3.2.1. Natural Grafts

In bone grafts, bone material is harvested and implanted to reconstruct significant bone damage [109]. In bone regeneration, four qualities are typically sought (osteogenesis, osteoinduction, osteoconduction, and osteointegration), but are not always required [110]. Allografts (from a genetically non-identical donor who is the same species as the recipient), and xenografts (the donor is not the same species as the recipient) only possess two of these qualities, lacking osteogenesis and osteoinduction [109]. Therefore, autografts (both recipient and donor are genetically identical) are considered the ‘gold standard’ of bone grafts as they have all four of the desired characteristics. However, the addition of inductive molecules in allografts and xenografts can compensate for the lack of osteointegration, as the mesenchymal stem cells arrive at the fracture site and can initiate the process of bone regeneration under certain conditions [4]. If during the process of fracture healing the bone site lacks cell migration to the target site, and does not possess the required number of progenitor cells that can potentially proliferate and differentiate to become osteoblasts and chondrocytes, then non-unions can occur [111]. 

The mesenchymal stem cell (MSC) population is believed to play an important role in the signalling of fracture healing, and therefore may be responsible for non-unions occurring [112]. Hernigou and Beaujean performed a study [113] that compared the number of MSCs in 35 non-union sites to the sizes of cell populations in bone marrow donors for the same sites. A difference was seen, as the non-union sites had a small stem cell population compared to the donors. Additionally, the iliac crest of patients also showed lower progenitor cell density [113]. If the lack of available progenitor cells in the target tissue is the key reason for abnormal fracture healing, further studies on stem cell incorporation into bone graft implantations for successful treatment of non-unions are certainly needed.

Different donor sites can be used for bone grafts, chosen based on the tissue’s strengths and weaknesses [109,114]. All three bone graft types have several drawbacks such as pain, high chance of site morbidity or immune response [115]. Therefore, research in recent years has also focused on advanced bone repair strategies and using optimal orthobiologics for tissue engineering [116]. 

#### 3.2.2. Conventionally Fabricated Scaffolds

Tissue engineering involves the use of scaffolds, growth factors and stem cells to accelerate bone regeneration. A family of growth factors located in the demineralized bone matrix, called bone morphogenetic proteins (BMPs), are particularly used in bone regeneration research, playing a major role in osteoinduction [117]. Other growth factors that induce bone regeneration include the transforming growth factor beta (T-GFβ) superfamily, insulin like growth factor 1 (IGF-1), fibroblast growth factors (FGFs), platelet-derived growth factors (PDGFs) and vascular endothelial growth factors (VEGFs) [118]. The mechanisms of growth factor targeting of the correct site through drug delivery, as well the evaluation of the appropriate dose of administration, are currently being researched [118]. Recently, it has been demonstrated that biomimetic peptides could be used instead of growth factors, as a cheaper and more straightforward option [119].

Bone graft substitutes (BGS) are being used as a scaffold component for tissue engineering when the natural healing of bones is hindered and a non-union occurs [120], which may occur from events such as infection or co-morbidities [115,121]. Designed to improve vascularization and mechanical strength, synthetic scaffolds allow the cells to reform new bone tissue whilst being functional in a biological environment and degrade naturally over time [6]. The synthetic scaffold may be deemed sufficient in treatment but, in more severe cases, molecules like growth factors or hormones may be used in addition, possibly supplemented by cell therapy [115]. Such approaches were reported to achieve the desired effects, improving healing rates during the use of scaffolds [122].

#### 3.2.3. Three-Dimensionally Printing Scaffolds

The use of computed tomography (CT) scans to make three-dimensional (3D) images of the patient’s anatomy allows for the manufacturing of 3D printed scaffolds. Bone grafts made using CT scans are more precise in their structure than grafts without it, leading to a more efficient return in function post-surgery. However, time is needed for the 3D scaffold to be made and it is not always possible to be manufactured in the same location as the surgery [115]. Types of 3D printing include powder-based, selective laser sintering (SLS), 3D plotting using ceramic cement, and fused deposition modelling (FDM) [122]. Strategies are also being developed to improve the critical process of post-surgery vascularization, including methods using stem cells [123,124]. The problem of inadequate vascularization can stem from using allografts that are slower in healing in the initial phase of implantation, with ingrowths possible before the blood vessels of the host come into contact with the implanted scaffold [124]. The larger the bone defect, the more likely vascularization problems are to occur, as a larger blood supply is required in the implant [7]. However, even with the best scaffold, vascularization, along with mechanical strength, can still be improved through the addition of factors mimicking all aspects of bones, including their mechanical, biological, mass transport and microstructure geometry properties [3]. It is crucial that scaffolds have a porous property to enhance vascularization [6]; 3D-printed scaffolds are commonly used due to the convenience of designing porous holes, which improves the biochemical properties of the implant [125]. On the other hand, rapid prototyping (RP) combined with additive manufacturing (AM) has become the best method to replicate irregular 3D and interconnected porous scaffolds [3]. The method of creating scaffolds through AM is carried out one layer at a time [125]. Computer aided software testing (CASTS) can be used to obtain an adequate structure for the scaffold, in addition to software such as computer-aided design (CAD) that aids intricate designs. However, even with the use of these aids, the performance of biomechanical and flow properties can be rather poor [3]. A way to improve flow properties is through increasing the surface–volume ratio of the scaffold [3]. One approach is implicit surface (IS) modelling that uses trigonometry to optimize this ratio for 3D scaffolds [3]. Delivery and more precise dose growth factors, an issue mentioned above, can be achieved via 3D-printed scaffolds [126]. Orciani et al. stated that angiogenesis might be deterred by the co-culture of stem cells, such as bone marrow mesenchymal stem cells (BM-MSCs) on an endothelial cell scaffold [127]. For differentiation, proliferation and cell migration to occur, the signalling cascades need a strong stimulus. The two populations of cells could reasonably weaken each other’s stimuli. Therefore, alternatives that improve angiogenesis such as porous nanotechnology scaffolds and growth factors could be used in conjunction with co-cultures to ensure bone formation is not limited. As 3D printing is reasonably priced and recent advances in this field allow researchers to locate bioprinters at the same site as the procedure, AM implants have great potential to be used in the mass market [128]. Midha et al. said that 3D bioprinting technology could be introduced in the next few years [129]. Nevertheless, they acknowledged that 3D printing still has some hurdles to manoeuvre, such as complex shaped grafts needing a higher resolution printer for precision than what is currently available. 

#### 3.2.4. CV-TEBG and Nanotechnology-Related Scaffolds

Another method that has been developed to help vascularization is tissue engineered scaffolds with a core-shell composite structure (centrally vascularized tissue engineering bone grafts; CV-TEBG) [7]. Made up of an angiogenic core and an osteogenic shell, the core-shell is designed to quickly establish itself in the vascular network and prevent central necrosis [7,123]. CV-TEBG uses a co-culture of mesenchymal stem cells and endothelial progenitor cells in collagen hydrogels that enhances angiogenesis and vascularization in the tissue core. Mesenchymal cells are also present in the osteogenic shell. A hollow cylinder β-tricalcium phosphate (β-TCP) scaffold provides the mechanical support and the ability to form new bone cells. β-TCP scaffolds are also porous and non-toxic to the host cells—essential qualities for their use in bone regeneration, as growth factors, stem cells and connective tissue can move and form inside the pores [130]. A study found that osteogenesis is improved in pores that are 1–2.5 mm in size, compared to pores that are only 1 mm wide [130]. Calcium phosphates are a commonly used material, based on their osteointegrative and osteoconductive characteristics. In the body, calcium phosphates form a hydroxyapatite layer, an essential ingredient for bone formation that enhances tissue integration [131]. A study that compared the suitability of β-TCP and bioglass combinations of scaffold materials found that in load bearing applications, the mechanical techniques were limited by 3D printing, despite their biocompatibility properties [131]. The review by Popov et al. gives the example of titanium 3D printed implant, admitting that the use of metal is a novelty in implantations and therefore there are a lot of questions needing to be answered before undergoing clinical trials [128]. A different study has found that silver (Ag), gold (Au) or a combination of the two metals could potentially be used as scaffolds if they are doped in hydroxyapatite nanoparticles [132]. Au has been used in biomedical applications for a while, and both the metals are commonly involved in the use of nanoparticles [133]. 

Nanotechnology is a further development for bone regeneration research, and nanoparticles are being increasingly used as orthobiologics [13]. Bone structure can be mimicked through the knowledge of its natural composition make-up of collagen fibrils and inorganic calcium phosphate crystals [134]. Hydroxyapatite nanoparticles are very similar to natural bone biomaterials [13]. Unfortunately, hydroxyapatite has a low solubility in the body and is therefore potentially harmful for the cell environment in the long term [130], leading to the consideration of alternatives that are more biocompatible, such as Au particles [13,135]. The osteogenic differentiation of HUVEC mesenchymal stem cells (hMSCs) may be enhanced using hydroxyapatite Au nanoparticles to activate the Wnt/β-catenin signalling pathway [13]. In contrast, nanotopography is a different use of nanotechnology for bone regeneration, allowing one to mimic the specific extracellular matrix environment of tissues [136]. Nanotopography describes the structural features such as nanopatterns, nanopores, or surface topography which enhance osteoinductivity and osteointegration of scaffolds [6]. Cell fate and differentiation are influenced by specific nanotopography structures [137]. Different lattices, made by repeats of identically sized and shaped cells, were tested by unfolding through the flattening of the cells. Then, 3D printing was used to demonstrate that lattices are able to refold themselves. Due to the lattices being highly malleable depending on their porosity, they can be designed to have unique functions. Techniques such as electron beam induced deposition (EBID) are used to introduce various nanopatterns on the surface of the lattice surface and obtain 3D shapes and various sized layers of lattices. These different ornaments can then be combined with lattice folding patterns, and potentially develop biomaterials to induce tissue regeneration by guiding stem cell differentiation. Therefore, stem cell lineage and differentiation can be guided by topographical cues, as well as chemical and mechanical stimuli [138]. 

In summary, bone regeneration treatments are an advancement from autografts, allografts and xenografts, preventing problems such as poor vascularization, pain and, in the latter two cases, autoimmune responses. The development of tissue engineering allowed stem cells and growth factors—such as BMPs—to be included in the scaffolds and accelerate the healing rates of the site. However, the mechanisms of growth factor action still need to be refined to ensure the correct dose and delivery to the target site. As an alternative to using growth factors, biomimetic peptides are being examined as a more suitable and cheaper material. The development of 3D printing has provided numerous improvements for scaffold mechanical structure porosity, being the first step to designing new treatment methods. Biomaterials such as β-TCP and hydroxyapatite Au nanoparticles show potential for use in bone scaffolds, improving bone regeneration through their osteogenic properties, as well as promoting tissue formation. An advantage of β-TCP is its similarity to the actual bone. However, B-TCP currently lacks mechanical strength, which limits its use in clinical trials. On the other hand, hydroxyapatite Au nanoparticles can also enhance stem cell activity but are seen as risky, as cell stress may be caused due to hydroxyapatite nanoparticles’ low solubility in the body. The advancements in nanotechnology have also led to the creation of nanotopography, which imitates the extracellular environment and can be used to create sophisticated lattices which influence cell differentiation lineage.

#### 3.2.5. Investigating New Therapeutic Targets

##### Secretory Proteins

Small signalling molecules may play a key role in the interactions between osteogenic cell types. RANKL protein was shown to mediate the differentiation of osteoclast precursors when secreted from neighbouring osteoblast cells [139]. Additionally, it was demonstrated that TGF-β1 released by osteoclasts during bone resorption induces the migration of MSCs to resorptive sites to differentiate into osteoblasts and form new bone, hinting at coupled mechanisms between osteoblasts and osteoclasts in maintaining and forming new bones [140]. The complicated nature of the interactions between osteoblasts, osteocytes, osteoclasts and resident progenitor cells in the human skeleton highlights a need for further study in the area, particularly important in potential implementation of osteogenic stem cell therapies.

Nuclear factor kappa-β ligand (RANKL) is a stimulatory protein during osteoclast maturation and is important for bone regeneration [141]. For bone grafts to promote healing, the regulation of this protein’s expression, particularly in aged patients, has a significant therapeutic potential. In a mouse study, Ito et al. found that RANKL gene expression was decreased after the application of an allograft, with the results then noting that healing and vascularisation was improved when RANKL and VEGF (transferred by recombinant adeno-associated virus) was added to the site [142]. The levels of osteoprotegerin (OPG) and receptor activator of nuclear factor kappa-β (RANK) can directly manipulate the activities of RANKL [143]. By itself, RANKL stops osteoclast activity. Meanwhile, OPG acts as an antagonist to RANKL activation, with the loss of bone mass being attributed to the increased expression of RANKL [144] and OPG shown, in the study by Jin et al., to overturn this effect. In ageing, it is thought that OPG downregulation, and RANKL upregulation is the cause for increased resorption [145]. In turn, high resorption is linked to the development of osteoporosis. A study by Tera et al. proposed that, in ageing, evasive parathyroid extract (PTE) slows down bone resorption and increases the expression of RANK [145]. This need for correctly balancing the levels of expression emphasises the importance of strategically implanting RANKL into bone scaffolds during future applications.

##### Signalling Pathways

The Wnt signalling pathway is known to play a key role in skeletal homeostasis, in particular osteoblast and chondrocyte differentiation [146]. In humans and some other mammals, there are 19 *Wnt* genes, each encoding a lipid-modified glycoprotein [147]. These WNT proteins interact with frizzled (FZD) cell surface receptors to activate intracellular pathways and regulate development across organisms. The key regulatory step is the inhibition of the AXIN1 complex, responsible for degradation of β-catenin, the downstream effector protein of the Wnt pathway [148].

It has also been suggested that WNT proteins act as paracrine factors through secretion in extracellular vesicles including exosomes [12]. The complex Wnt pathway is believed to be important for osteogenic differentiation, as loss-of-function mutations in low-density lipoprotein receptor-related protein 5 (LRP5—a co-receptor of WNT) were found to cause osteoporosis-pseudoglioma syndrome, while gain-of-function mutations in LRP5 caused osteosclerosis [146,149]. These conditions are characterised by abnormally low, or high bone density respectively.

The relationship between the regulation of the Wnt pathway and osteoblast differentiation could prove relevant to researching new methods of bone treatment. The promotion of Wnt signalling in mice by the introduction of the L-WNT3A protein encouraged autograft healing potential [105]. WNT antagonists such as Dickkopf-related protein 1 and sclerostin could also be inhibited to encourage osteoblast differentiation [147]. Sclerostin, encoded by the *SOST* gene, and Dickkopf-related protein 1, encoded by the *DKK1* gene, can both inhibit the Wnt signalling pathway through binding to the LRP5/6 co-receptors [150,151]. 

Exosomes released from neighbouring cells can transfer genetic information such as miRNAs, as well as proteins such as WNTs and thereby regulate cell signalling, influencing the cell fate of precursor cells [12,152]. Approximately one third of the human genome is regulated by microRNAs (miRNAs). These non-coding RNAs, of which over 2000 have been described in humans, each regulate the expression of hundreds of genes by binding mRNA prior to translation and encouraging the degradation of the mRNA. The development of miRNAs as therapeutic treatments involves the production of a mimic in cases of diseases caused by miRNA dysfunction, or an inhibitor in the cases of diseases with abnormally high levels of miRNAs [153]. Several miRNAs have already been linked to osteogenic differentiation.

Apart from regulating differentiation in precursor cells, miRNAs can also stabilize a pluripotent state after cell dedifferentiation into induced pluripotent stem cells (iPS) by inhibiting cell lineage-specific transcription factors. Once a cell commits to differentiating again, new miRNAs are induced to inhibit the transcription factors specific for maintaining the pluripotent state, such as *Sox2, Oct4* and *Nanog* [154].

In an in vitro model, miRNA-27a was shown to regulate osteoblast differentiation and bone apoptosis in arthritis, the effects of which were closely tied to *PPAR-γ* expression [155]. Another study linking miRNA-27a to osteoblast differentiation and inhibition of adipogenesis indicated *PPAR-γ* and *GREM1* as potential direct targets, and suggested a therapeutic possibility in cases of osteonecrosis of the femoral head [156]. miRNA-365a-3p and miRNA-217 were found to bind to RUNX2 and thereby inhibit osteogenic differentiation [157,158]. Senescent endothelial cells secrete miRNA-31 to neighbouring MSCs via extracellular vesicles, inhibiting osteogenic differentiation by binding to FZD3 [159].

The complicated nature of these interactions highlights an area for further research, as miRNAs interact with regulatory gene products and hold potential to activate/inactivate the signalling pathways associated with cell differentiation. Exosomes derived from osteoblast were shown to alter miRNA profile of neighbouring MSCs, thereby activating Wnt signalling and increasing β-catenin expression, resulting in differentiation to osteoblast cells [152].

## 4. Section III: Application of Osteogenic Stem Cells in Animal Model Research and Human Clinical Trials

### 4.1. Direct Administration of ASCs and MSCs

The therapeutic evaluation of stem cells in allogenic models requires the evaluation of animal models prior to and during treatment as a means of assessing the homing properties of cells in response to local transplantation. The usefulness of cells with osteogenic potential generates clinically encouraging cell-based treatments applied to complex fractures in bone defects and bone diseases like osteonecrosis, osteoarthritis, osteosclerosis, ovariectomy-induced osteoporosis and a few genetic disorders, including hypophosphatasia and achondroplasia [17,160]. Even though bone marrow stromal cells (BMSCs), and adipose tissue-derived stem cells (ASCs) have been demonstrated to hold promising osteogenic capacity; only recently a growth has been observed in the number of applications utilizing live BMSCs and ASCs in preclinical research. BMSCs, including mesenchymal stromal cells (MSCs) and amniotic stromal cells (AMSCs), not only inhibit the proliferation of allogenic T cells and are negative for most immunological surface markers, but also retain immune modulating properties even after osteogenic induction in vitro [103,161]. In general, allogeneic settings including paracrine factors, transdifferentiation and immunomodulation are aided by the absence of HLA class II receptors, which induce the expression of regulatory T cells and therefore suppress the immune response to the rejection of allogenic grafts, on MSCs [162]. The injection of either ASCs or MSCs may reduce chances of acute transplant rejection, as intra-articular injections of micro-fragmented adipose tissue to treat for cartilage defect in mice displayed successful repair of defects and consequently normal cartilage formation [16]. In human clinical trials, the condition of infants with severe hypophosphatasia was improved through the transplantation of ex vivo expanded allogeneic MSCs obtained from the relatives of the patients [163]. Recently, another study by Alfotawi et al. described a new procedure in bone engineering using adipose tissue to enable the interconversion of mesenchymal stem cells from adipocytes to osteocytes. In other words, supporting associated bone formation and balancing adipogenic interconversion creates future possibilities to upregulate osteogenesis in osteoporosis, osteonecrosis and osteoarthritis [15]. The ongoing preclinical research mostly focuses on application of stem cells with the highest osteogenic capacity in human clinical trials. 

### 4.2. Bone Marrow Mononuclear Cells and Bone Marrow Concentrate 

Currently, to treat bone pathologies, transplant therapies using osteogenic autologous bone grafts are being replaced with patient-specific cell therapies using autologous bone marrow mononuclear cells (BM-MNCs) consisting of stem cells, dendritic cells, monocytes and lymphocytes [164]. In addition, in all cases of patients suffering from long-bone pseudoarthrosis, complete bone consolidation was achieved when treated with allogenic cancellous bone grafts combined with iliac crest-derived autologous BM-MNCs [164]. Interestingly, comparison of osteogenic ability of rat bone marrow concentrate (BMC) and mesenchymal stem cells suggests that BMC has higher osteogenic potential, due to the cross talk of hematopoietic stem cells, mesenchymal stem cells and osteoprogenitors, which does not occur in therapies based solely on MSCs [17]. Bone marrow aspirate used in the treatment of osteonecrosis during the minimally invasive decompression of the femoral head halted the progression of the disease and resulted in pain relief [165]. Analogically, delayed unions existing after femoral lengthening surgeries in achondroplastic dwarfs were improved by adjuvant in-situ therapy performed using injections of bone marrow concentrate, with complete recovery taking 2–10 months in all patients [166]. Nonetheless, a study aiming to enhance the biomechanical properties of distraction osteogenesis (DO) by supplementation of distracted bone with bone marrow aspirate concentrate (BMAC) delivered non-significant results and failed to improve bone strength [167]. Intravenously injected BMC, combined with iloprost, improved fracture healing in patients with avascular necrosis of the femoral head compared to treatment with core decompression alone [168]. Six year post-operative outcomes of proceeding concentrated autologous bone marrow aspirate transplantation (CABMAT) resulted in the successful prevention of femoral head collapse [169]. Clinical evaluation of the spared joints in autologous concentrated bone marrow grafting conducted on four patients affected by advanced osteonecrosis resulted in recovery and improved motion [170]. The yearlong human model study by De Riu et al. demonstrated efficacy of bone marrow nucleated concentrate (BMNc) in patients with degenerative temporomandibular joint disorders (TMDs). Patients receiving intra-articular BMNc injections showed improved chewing and maximum interincisal opening, but most importantly reported significant pain relief [171]. 

In summary, there is a wide range of potentially useful treatments based on bone marrow-derived stem cells, currently or soon to be widely explored in human clinical trials. These studies should bring significant benefits to regenerative medicine, due to the great osteogenic capacity of mesenchymal and adipose-derived stem cells. It is highly possible that in the future, researchers working on the improvement of methods delivering promising results in the fields of stem cell and genetic therapies will focus on exploring the application of stem cells injected in the form of bone marrow concentrate. However, despite the significant therapeutic capacity of BMC, the purpose of its delivery still needs to be addressed. Genetic reprogramming to invert already-differentiated stem cells is coming up as an alternative to stem cell therapies treating disorders such as osteoporosis. 

An overview of current clinical trials of bone disease therapies based on MSCs and ASCs, alone or combined with other factors, was compiled and presented in Table 1. 

## 5. Conclusions

Overall, modern medical challenges, mostly associated with the constantly extending lifespan of the population, definitely include new ways of treating a range of skeletal system-associated diseases. The results of a range of in vitro, animal and human studies indicate that ASCs and MSCs, alone or with combination with other therapies, could become the basis of a novel clinical approach, improving the outcomes of bone disease treatment, as well as limiting the adverse side effects. Due to the high complexity of the stem cell-associated processes, a large amount of research is still being performed to fully grasp the complete molecular mechanisms governing the processes of ASC and MSC proliferation and differentiation and their exact role in the processes of bone regeneration. Nevertheless, a significant number of clinical trials are currently being conducted or have been completed (with a promising degree of success), further supporting the potential involvement of these cells in regenerative and reconstructive medicine in the 21st century.

## Figures and Tables

**Table 1:** An overview of the recently completed clinical trials that employed ASCs and MSCs in treatment of bone related diseases. All studies can be found in the Clinical Trials database (https://clinicaltrials.gov/) using references provided.

**Completed Research Studies and Clinical Trials on Bone Regeneration and Reconstruction**			
**Study Title**	**Conditions**	**Interventions**	**Principal Investigator (Year of Study)**
Clinical Trial of Intravenous Infusion of Fucosylated Bone Marrow Mesenchyme Cells in Patients with Osteoporosis	Osteoporosis Spinal fractures	Biological: Fucosylated MSC for Osteoporosis	Lonano et al. (2018)
A New Procedure in Bone Engineering Using Induced Adipose Tissue	Osteogenesis Osteonecrosis Osteoarthritis	Interconversion of mesenchymal stem cells from adipocytes to osteocytes	Alfotawi et al. (2019)
Repair of long-bone pseudoarthrosis with autologous bone marrow mononuclear cells combined with allogenic bone graft	Long-bone Pseudoarthrosis	Allogenic grafts supplementation with autologous BM-MNCs	Fernandez-Bances et al. (2013)
Use of concentrated bone marrow aspirate and platelet rich plasma during minimally invasive decompression of the femoral head in the treatment of osteonecrosis.	Osteonecrosis	Surgical procedure including decompression of femoral head and injection of mesenchymal stem cells	Martin et al. (2013)
Clinical Trial of Allogenic Adipose Tissue-Derived Mesenchymal Progenitor Cells Therapy for Knee Osteoarthritis	Osteoarthritis	Mesenchymal progenitor cells	Zhao X et al. (2018)
Bone Marrow Aspirate Concentrate in Combination with Intravenous Iloprost Increases Bone Healing in Patients With Avascular Necrosis of the Femoral Head: A Matched Pair Analysis.	Avascular necrosis of the femoral head	Intravenous injection of bone marrow concentrates in association with iloprost	Pilge et al. (2017)
Reduced Intensity AlloTransplant for Osteopetrosis	Osteopetrosis	Stem Cell or Umbilical Cord Blood Transplantation	Paul Orchard, MD (2008)
Treatment of osteonecrosis of the femoral head by core decompression and implantation of fully functional ex vivo-expanded bone marrow-derived mesenchymal stem cells: a proof-of-concept study	Osteonecrosis of the Femoral Head	Bone marrow aspirate	Mardones et al. (2019)
Inefficacy of autologous bone marrow concentrate in stage three osteonecrosis: a randomized controlled double-blind trial	Osteonecrosis	Bone marrow concentrate	Hauzeur et al. (2018)
The Effects of Intra-articular Injection of Mesenchymal Stem Cells in Knee Joint Osteoarthritis	Osteoarthritis	Mesenchymal stem cells	Mohsen Emadeddin, MD (2015)
Treatment of knee osteoarthritis with autologous mesenchymal stem cells: A pilot study	Osteoarthritis	Mesenchymal stem cells	Orozco et al. (2013)
Intra-articular Injection of Mesenchymal Stem Cells in Osteonecrosis of the Femoral Head	Osteoarthritis	Placenta Derived Mesenchymal Stem Cell	Yu tang et al. (2018)
Risk-Adapted Allogeneic Stem Cell Transplantation for Mixed Donor Chimerism In Patients With Non-Malignant Diseases	Bone Marrow Failure	Fludarabine, Cyclophosphamide Cyclophosphamide 40, Cyclophosphamide 30	Garvin et al. (2011)
Safety Study of Gene Modified Donor T-cells Following TCR Alpha Beta Depleted Stem Cell Transplant	Osteopetrosis	Biological: BPX-501 T cells Drug: rimiducid	Locatelli et al. (2019)
Use of Cell Therapy to Enhance Arthroscopic Knee Cartilage Surgery	Bone fracture	Standard microfracture arthroscopic surgery, autologous stem cell administration	Burke et al. (2019)
T-cell Depleted Alternative Donor Transplantation	Bone Marrow Failure Osteopetrosis	CliniMACS^®^ (T cell depletion)	Gilman et al. (2017)
Clinical Trial of Intravenous Infusion of Fucosylated Bone Marrow Mesenchyme Cells in Patients with Osteoporosis	Osteoporosis	Fucosylated MSC for Osteoporosis	Linares Ferrando et al. (2017)
Hip preserving surgery with concentrated autologous bone marrow aspirate transplantation for the treatment of asymptomatic osteonecrosis of the femoral head: retrospective review of clinical and radiological outcomes at 6 years postoperatively	Femoral head collapse	Surgical procedure including autologous bone marrow aspirate transplantation	Tomaru et al. (2017)
Treatment of Atrophic Non-union by Pre-osteoblast Cells	Non-union of long bone	percutaneous autologous pre-osteoblast cells implantation	Hauzeur et al. (2012)
Autologous Stem Cell Therapy for Fracture Non-union Healing	Non-union healing	carrier plus in vitro expanded autologous BMSCs	Richardson et al. (2014)
Cell Therapy by Autologous BMC for Large Bone Defect Repair	Large bone defect	Bone marrow concentrate beta-TCP Chronos^®^ Synthes	Marzi et al. (2019)
Cell Therapy by Bone Marrow-derived Mononuclear Cells (BMC) for Large Bone Defect Repair: Phase-I Clinical Trial	Large bone defect	Bone marrow concentrate	Marzi et al. (2016)
Treatment of Atrophic Non-union Fractures by Autologous Mesenchymal Stem Cell Percutaneous Grafting	Non-union fractures of bone	Administration of Mesenchymal Stem Cells Culture medium without MSC	Hauzeur et al. (2013)
Autologous BM-MSC Transplantation in Combination with Platelet Lysate (PL) for Non-union Treatment	Non-union fractures of bone	Percutaneous injection	Aghdami et al. (2015)
Pulse Shortwave Therapy in Cervical Osteoarthritis	Osteoarthritis	ActiPatch Etoricoxib 60 mg	Mohammad et al. (2019)
Safety and Efficacy of Meloxicam Compared to Other Nonsteroidal Anti-inflammatory Drugs (NSAIDs) in an Observational Cohort Study of Patients with Rheumatoid Arthritis, Osteoarthritis, Lumbago, Scapulohumeral Periarthritis, Neck, Shoulder and Arm Syndrome	Osteoarthritis	Non-Steroidal Anti-Inflammatory Drugs (NSAIDs) except etodolac	Boehringer Ingelheim (2014)
Investigation of Mesenchymal Stem Cell Therapy for the Treatment of Osteoarthritis of the Knee	Osteoarthritis	BMAC injection	Ruane et al. (2019)
**Ongoing Research Studies and Clinical Trials on Bone Regeneration and Reconstruction**			
**Study Title**	**Conditions**	**Interventions**	**Principal Investigator (Study Start Year)**
Stem Cell Recruitment in Osteoporosis Therapy	Low Bone Density	Teriparatide, Alendronate, calcium and vitamin D	Suzanne Jan De Beur, MD (2012)
Phase 2a Study on Intravenous Infusion of Autologous Osteoblastic Cells in Severe Osteoporosis	Severe Osteoporosis	PREOB^®^ Intravenous Infusion	Anderlecht, Belgium (2019)
Mesenchymal Stem Cells in Osteonecrosis of the Femoral Head	Avascular Necrosis of Femur Head	XCEL-MT-OSTEO-ALPHA	Màrius Aguirre et al. (2019)

## Abbreviations

ALP—alkaline phosphatase, AM—additive manufacturing, AMSCs—amniotic stromal cells, AP-1—Activator protein 1, BCA1—B cell-attractive chemokine, BGS—bone graft substitutes, BMAC—bone marrow aspirate concentrate, BMC—bone marrow concentrate, BM-MNCs—bone marrow mononuclear cells, BMNc—bone marrow nucleated concentrate, BMP2—bone morphogenetic protein 2, BMSCs—bone marrow stem cells, BSP—bone sialoprotein, β-TCP—Beta-tricalcium phosphate, CABMAT—concentrated autologous bone marrow aspirate transplantation, CAD—Computer-Aided Design, CAST—computer aided software testing, CatK—Cathepsin-K, CT—computed tomography, CV-TEBG—centrally vascularized tissue engineering bone grafts, CXCR4—C-X-C chemokine receptor type 4, Dlx5—distal-less homeobox 5, EBID—electron beam induced deposition, EGFR—epidermal growth factor receptor, ENP1—Ectonucleotide pyrophosphatase/phosphodiesterase 1, Erk—extracellular receptor kinase, FDM—fused deposition modelling, FGF—Fibroblast Growth factor, Fra—Fos related antigen, FZD—Frizzled, HDAC—histone deacetylase, HGF—hepatocyte growth factor, hMSCs—HUVEC mesenchymal stem cells, HUVEC—human umbilical vein endothelial cells, IGF-1—insulin like growth factor, Ig-like—immune globulin like, IFN-γ—interferon-γ, IL-17—interleukin 17, iPS—induced pluripotent stem cells, IS—implicit surface, JNK—jun N-terminal Kinase, LRP5—low-density lipoprotein receptor-related protein 5, MAP—mitogen-activated protein, MHC—Major histocompatibility complex, MITF—Microphthalmia-associated transcription factor, MSC—mesenchymal stem cells, MSCA-1—mesenchymal stem cells antigen-1, NFATC1—nuclear factor of activated T cells, NK-KB—nuclear factor kappa-light-chain-enhancer, OPG—osteoprotegerin, OSCAR—Osteoclast-associated immunoglobulin-like receptor, Osx—Osterix/Sp7, PDGFs—platelet-derived growth factors, PDPCs—Periosteum derived progenitor cells, PGE2—prostaglandin E2, PI3K—phosphoinositide 3-kinase, Prx1—Peroxiredoxin, PTE—parathyroid extract, RANK—nuclear factor kappa-beta, RP—rapid prototyping, Runx2—runt related transcription factor 2, SDF-1—stromal cell-derived factor 1, SLS—selective laser sintering, TNF—Tumor necrosis factor, TRAF6—TFN receptor associated factor, TRAP—Thrombin receptor-activating peptides, VEGF—Vascular endothelial growth factor.
